# Obstructive Sleep Apnea Awareness Among Dentists in Saudi Arabia: A Cross-Sectional Study

**DOI:** 10.7759/cureus.36463

**Published:** 2023-03-21

**Authors:** Nozha Sawan, Heba Bakhsh, Mohammed Aldossary, Reema Alhussan, Nourah Alharbi, Hoda M Abdellatif

**Affiliations:** 1 Department of Preventive Dental Sciences, College of Dentistry, Princess Nourah Bint Abdulrahman University, Riyadh, SAU; 2 General Directorate of Research and Studies, Ministry of Health, Riyadh, SAU; 3 Dentistry, College of Dentistry, Princess Nourah Bint Abdulrahman University, Riyadh, SAU; 4 Public Health Sciences, College of Dentistry, Texas A&M University, Dallas, USA

**Keywords:** oral appliances, orthodontics, oral health, dental care, practice, knowledge, dentists, saudi arabia, obstructive sleep apnea

## Abstract

Objectives: This study aims to assess the knowledge and clinical practices among dentists in Saudi Arabia regarding obstructive sleep apnea (OSA).

Material and methods: This cross-sectional study was conducted over a period of 8 months across the whole country. A self-administered questionnaire was used to assess the OSA knowledge and practices. The developed questionnaire was closed-ended questions and consisted of three parts: (1) demographic information such as age, gender, work experience, workplace, and specialty; (2) knowledge of OSA; and (3) OSA management practice. The knowledge score was calculated based on six questions, each of which gives one point when answered correctly.

Results: A total of 450 dentists from all over the country were surveyed, with a predominance of females (55.6%) and Saudis (59%). About 56% learned about OSA in their undergraduate study, with theoretical lectures being the main source of information. The overall knowledge score was 3.09, with specialists having a higher score than general practitioners (GPs) (p<0.001) and those who learned about OSA in their post-graduate study having higher scores (p<0.001). Despite this, 58.89% never asked their patients about sleep history, and only 19.11% diagnosed patients with OSA before. Only 7.56% used oral appliances to treat OSA, and only one-quarter referred patients to a physician for a sleep-disordered diagnosis. However, 79.33% were willing to attend a continuing education course on managing OSA patients.

Conclusion: Our study shows that dentists in Saudi Arabia lack fundamental understanding regarding OSA and their role in screening, diagnosing, and treating patients with OSA, highlighting the need to educate the dental fraternity. Dental GPs and specialists, including orthodontists, must be actively involved in diagnosing and treating this life-threatening health issue.

## Introduction

Obstructive sleep apnea (OSA) is characterized by recurrent partial or complete obstruction of the upper airway during sleep [1]. Insomnia, hypercapnia, hypoxia, and snoring are the main features of OSA [2,3]. Depression, cognitive impairment, cardiovascular disease, diabetes, and hypertension are some health complications that may develop due to untreated OSA [4]. OSA is associated with daytime sleepiness, anxiety, fatigue, and a higher risk of traffic accidents and functional impairment [2-5]. Marital and social issues might arise as a result of snoring, making it a serious problem that requires immediate management. Obesity is a significant risk factor for OSA [1]. Orofacial structural anomalies, such as enlarged uvula, hypertrophy of palatine tonsils, macroglossia, and mandibular micrognathia, as well as periodontal disease, alcohol intake, tobacco use, family history, and advanced age, have all been linked to OSA [6]. Because of expensive diagnostic testing and the lack of awareness, most people with OSA do not recognize their condition [3]. Another reason may be the inadequate education provided to subjects with OSA by their dentist or physician.

Early diagnosis is an essential component in effective patient management, and a thorough clinical examination using a simple questionnaire is a fundamental part of this process [4]. In order to confirm a diagnosis of OSA, polysomnography (PSG) remains the diagnostic gold standard [7]. Polysomnography, also called a sleep study, is a comprehensive test used to diagnose sleep disorders allowing overnight monitoring of oxygen saturation and respiration rates during sleep is required [8,9]. At least five apneas or hypo apneas per hour, leading to sleep fragmentation and a drop in blood oxygen saturation, are diagnostic of OSA [10].

Sleep apnea can be effectively managed in a number of ways based on an accurate diagnosis. Previous studies demonstrated the effectiveness of basic, modern imaging methods for early diagnosis of OSA [1,4,11]. The intensity of symptoms, the presence or absence of clinical consequences, and the underlying cause of upper airway obstruction all play a role in determining how OSA is treated [12]. Continuous positive airway pressure (CPAP) has been the treatment of choice; however, patient compliance is not optimal [13]. Oral appliances were originally used to treat glossoptosis in 1900 by Pierre Robin; subsequently, the same appliance was adapted for use in treating sleep problems [14]. Oral appliances have recently been approved for the treatment of mostly snoring and mild to moderate OSA by the American Academy of Sleep Medicine (AASM) [15,16].

Although the guidelines published by the AASM and the American Academy of Dental Sleep Medicine state that dentists are not qualified to diagnose OSA [17], dentists still have an important role in identifying patients with OSA. Identifying, counseling, referring, and treating patients with OSA are all areas in which dentists contribute and, consequently, help reduce the problem of underdiagnosis of OSA [18]. Furthermore, dentists are the ones who confirm the appropriateness of oral appliance therapy and initiate the treatment.

A growing number of people in Saudi Arabia are being diagnosed with sleep apnea. The frequency of OSA in the Saudi Arabian population has been reported to be 39% in females and 33.3% in men [19,20]. Both the education of both physicians and dentists would have an impact on the early diagnosis and treatment of OSA. Therefore, one needs to understand the ability of Saudi dental professionals to recognize OSA patients effectively at an early stage. This study aimed to assess the knowledge and clinical practices among dentists in Saudi Arabia regarding OSA.

## Materials and methods

Study design and participants

A survey of dental practitioners in Saudi Arabia was conducted to assess the OSA's current knowledge and practice among dentists in Saudi Arabia. A convenient sample of Saudi dentists working in Saudi Arabia was invited to participate in the study through an e-mail using the Ministry of Health database. Ethical approval to conduct the study was obtained from the institutional review board of Princess Nourah Bint Abdulrahman University (PNU) (IRB # 21-0031). Because it is a self-administered questionnaire, no written informed consent was needed. Consent is implied by responding and completing the questionnaire.

Data collection

A self-administered questionnaire was used to assess the OSA knowledge and practices. The developed questionnaire was closed-ended questions and consisted of three parts: (1) demographic information such as age, gender, work experience, workplace, and specialty; (2) knowledge of OSA; and (3) OSA management practice. The content validity of the questionnaire was tested by presenting it to specialists from the College of Dentistry, PNU, and accordingly, modifications were made. The questionnaire was also pretested for wording and clarity. Instructions on how to fill out the questionnaire were provided, and it was made clear that participation was entirely voluntary. An online survey using Google Forms (Google Forms, 2019; a free web-based survey generator) was distributed among the targeted population. After the first 100 responses, the validity and reliability tests were assessed, resulting in a Cronbach's alpha of 0.81. The knowledge score was calculated based on six questions, each of which gives one point when answered correctly.

Data analysis

The SPSS Statistics version 24 (IBM Corp. Released 2016. IBM SPSS Statistics for Windows, Version 24.0. Armonk, NY: IBM Corp.) was used to code and analyze the data. The mean and standard deviation (SD) were used to describe continuous data (knowledge score). Categorical data, such as age group, gender, specialty, etc., were presented as frequencies and percentages. Comparing contentious variables was done using the student t-test and the ANOVA test, followed by Tukey post hoc analysis. If the p-value was lower than 0.05, the result was deemed significant.

## Results

Demographic characteristics

We obtained 450 responses from dentists all over the country out of a total of 804 participants, resulting in a response rate of 56%. Of the respondents, 55.6% were female and 59% were Saudis. The majority of respondents (60%) were aged between 24 and 35 years, followed by 36-45 years (25.1%), 46-55 years (9.6%), and >55 years (5.3%). Approximately 58% of the respondents were general practitioners (GPs), and 42% were specialists. Specialists of orthodontics (20.63%), prosthodontics (16.93%), restorative and esthetic dentistry (15.87%), and endodontics (14.81%) constituted the majority of respondents. Regarding the working sector, 46.89% were working in the governmental sector, 47.56% in the private sector, and 22.22% in the academic sector. About 53.6% of the participants had more than five years of experience, as shown in Table 1.

**Table 1:** Demographic characteristics of the participants GP: general practitioner; PNU: Princess Nourah bint Abdulrahman University; KSU: King Saud University; REU: Riyadh Elm University; KAU: King Abdulaziz University; IAU: Imam Abdulrahman Bin Faisal University

Parameters		N (%)
Age	24-35	270 (60%)
	36-45	113 (25.1%)
	46-55	43 (9.6%)
	>55	24 (5.3%)
Gender	Male	200 (44.4%)
	Female	250 (55.6%)
Specialty	GP	261 (58%)
	Specialized	189 (42%)
Field of specialty	Advanced general dentistry	5 (2.65%)
	Endodontic	28 (14.81%)
	Oral and maxillofacial surgery	18 (9.52%)
	Oral Medicine	9 (4.76%)
	Oral Pathology	2 (1.06%)
	Orthodontics	39 (20.63%)
	Pedodontics	11 (5.82%)
	Periodontics	18 (9.52%)
	Prosthodontics	32 (16.93%)
	Public health	4 (2.12%)
	Radiology	4 (2.12%)
	Restorative and Esthetic Dentistry	30 (15.87%)
Country of specialty	Saudi Arabia	111 (59%)
	International	77 (41%)
Bachelor's degree	PNU	77 (17.1%)
	KSU	119 (26.4%)
	REU	62 (13.8%)
	KAU	11 (2.4%)
	IAU	69 (15.3%)
	Missing	112 (24.9%)
Work sector	Private	214 (47.56%)
	Governmental	211 (46.89%)
	Academic	100 (22.22%)
Years of experience	<5	209 (46.44%)
	5-10	107 (23.78%)
	11-15	54 (12%)
	>15	80 (17.78%)

Knowledge of OSA

Fifty-six percent of the respondents learned about OSA when they were undergraduates, 36% during their post-graduate study, and 8% after post-grad. The main source of information was theoretical lectures (69.78%), workshops and conferences (35.33%), training and simulation (30.22%), and group discussions and seminars (7.78%). When they were asked about the gold standard for diagnosis of OSA, 48.4% chose PSG, 24.2% chose case history, and 17.1% chose questionnaires. In terms of the diagnostic features, a full night at-home sleep study (51.33%) and physical examination  (55.56%) were the most frequent answers among the respondents. From the respondents' point of view, the main symptoms of OSA were loud snoring (71.33%), gasping for air during sleep (68.00%), excessive daytime sleepiness (50.22%), morning headache (45.11%), awakening with a dry mouth (36.22%), loss of concentration (28.00%), difficulty staying asleep (24.44%), and choking (7.11%). The risk factors of OSA were weight (81.11%), age (41.11%), gender (25.78%), and pneumonia (31.56%). In terms of the gold-standard treatment of OSA, only 36.44% chose CPAP, and 19.56% chose oral appliances. When they asked about who can provide the patients with oral appliances, 29.33% chose dentists, 15.67% chose physicians, and 37.78% chose both, as shown in Table 2.

**Table 2:** Knowledge questions and answers PSG: polysomnography; CPAP: continuous positive airway pressure

Questions	Answers	N (%)
At which of the following educational level were you introduced to the term "obstructive sleep apnea"	Undergraduate	252 (56%)
	Post-graduate	162 (36%)
	After post-grad	36 (8%)
How were you introduced: What was the instruction method used?	Theoretical lectures	314 (69.78%)
	Group discussion/seminar	35 (7.78%)
	Training and simulation	136 (30.22%)
	Workshops and conferences	159 (35.33%)
What is the gold standard for diagnosis of OSA?	Questionnaires	77 (17.1%)
	PSG	218 (48.4%)
	Case history	109 (24.2%)
	Do not know	46 (10.2%)
Which of the following are the symptoms of OSA? (You can choose more than one)	Loud snoring	321 (71.33%)
	Excessive daytime sleepiness	226 (50.22%)
	Morning headache	203 (45.11%)
	Gasping for air during sleep	306 (68%)
	Awakening with a dry mouth	163 (36.22%)
	Skin rash	17 (3.78%)
	Loss of concentration	126 (28%)
	Difficulty staying asleep	110 (24.44%)
	Choking	32 (7.11%)
	Fever	0 (0.0%)
	Do not know	19 (4.22%)
Which of the following is a diagnostic feature for OSA?	Blood sample	17 (3.78%)
	Chest X-ray	81 (18%)
	Full night in-lab sleep study	163 (36.22%)
	Full-night at-home sleep study	231 (51.33%)
	Physical examination	250 (55.56%)
	Family medical histories	158 (35.11%)
	Do not know	182 (40.44%)
Which of the following is considered a risk factor for OSA?	Weight	365 (81.11%)
	Age	185 (41.11%)
	Gender	116 (25.78%)
	Pneumonia	142 (31.56%)
	Do not know	28 (6.22%)
Which of the following is the gold-standard treatment for sleep apnea?	CPAP	164 (36.44%)
	Oral appliances	88 (19.56%)
	Surgery	60 (13.33%)
	Behavioral changes	28 (6.22%)
	Medications	10 (2.22%)
	Position therapy	30 (6.67%)
	Do not know	70 (15.56%)
Who can prescribe oral appliances for OSA patients?	Dentists	132 (29.33%)
	Physician	70 (15.56%)
	Both	170 (37.78%)
	Do not know	77 (17.11%)

Knowledge score

The overall knowledge score was 3.09±1.36. The knowledge score did not significantly differ between the respondents in terms of their age (p=0.359), gender (p= 0.987), the location of post-graduate study (p=0.794), the university of bachelor's degree (p=0.054), work sector (p=0.892), or years of experience (p=0.063). On the other hand, specialists had a higher knowledge score compared to GPs (3.44±1.88 vs. 2.83±1.67; p<0.001), respectively. In addition, orthodontic specialists had a significantly higher knowledge score compared to specialists of restorative and esthetic dentistry (3.94±1.29 vs. 2.72±1.39; p=0.016). The participants who learned about OSA during their post-grad study had higher knowledge scores than those who learned during undergraduate study or after post-grad (3.42±1.32 vs. 2.97±1.33 vs. 2.38±1.33; p<0.001), respectively. Moreover, participants who had previous experience in diagnosing or treating OSA cases had significantly (p<0.001) higher knowledge compared to those who did not diagnose or treat such cases before. The score was also higher in those who received previous training compared to others (p<0.001), as shown in Table 3.

**Table 3:** Knowledge score (*) significantly different in terms of knowledge score PNU: Princess Nourah Bint Abdulrahman University; KSU: King Saud University; REU: Riyadh Elm University; KAU: King Abdulaziz University; IAU: Imam Abdulrahman Bin Faisal University

Parameters		Mean± SD	P-value
Overall Knowledge Score		3.09±1.36	
Age	24-35	3.01±1.39	0.359
	36-45	3.21±1.33	
	46-55	3.07±1.33	
	>55	3.42±1.18	
Gender	Male	3.09±1.9	0.987
	Female	3.09±1.81	
Specialty	GP	2.83±1.67	<0.001
	Specialized	3.44±1.88	
Post-graduate study	Saudi Arabia	3.41±2.1	0.794
	Internationally	3.47±1.57	
Field of specialty	Orthodontics*	3.94±1.29	0.016
	Pedodontics	3.80±1.40	
	Periodontics	3.43±1.28	
	Restorative and esthetic dentistry*	2.72±1.39	
	Prosthodontics	3.41±1.21	
	Public health	2.67±0.58	
	Advanced general dentistry	2.00±1.41	
	Oral and maxillofacial surgery	3.88±1.52	
	Endodontic	3.62±1.28	
	Oral medicine	3.66±0.82	
	Radiology	3.00±0.81	
Bachelor's degree	PNU	3.03±1.31	0.054
	KSU	3.22±1.21	
	REU	2.80±1.21	
	KAU	2.45±1.21	
	IAU	2.74±1.51	
Work sector	Private	3.08±1.34	0.892
	Governmental	3.08±1.34	
	Academic	2.98±1.52	
Years of experience	<5	2.96±1.38	0.063
	5-10	3.36±1.34	
	11-15	2.93±1.29	
	>15	3.16±1.34	
At which of the following educational level were you introduced to the term "obstructive sleep apnea"	Undergraduate	2.97±1.33	<0.001
	Post-graduate	3.42±1.32	
	After post-grad	2.38±1.33	
Have you ever diagnosed an OSA patient?	Yes	3.76±1.48	<0.001
	No	2.93±1.79	
Have you treated an OSA patient?	Yes	3.8±1.89	<0.001
	No	3.01±1.79	
Have you ever fabricated any oral appliance for treating your patient with OSA?	Yes	3.21±2.53	0.60
	No	3.08±1.79	
Have you ever attended any continuing education courses on the management of OSA patients?	Yes	3.48±1.86	<0.001
	No	2.9±1.73	

Practice

The majority of respondents never asked their patients about their sleep history after observing the attrition of teeth in their mouths (58.89%). Similarly, about 63.56% never screened their patients for OSA, even if they had a history of snoring. Moreover, only 19.11% of the participants diagnosed patients with OSA; 52.33% of them diagnosed less than five cases per year. Approximately 9.78% of the respondents treated OSA cases; 38.64% of them treated more than five cases per year. In terms of oral appliances, only 7.56% of the respondents used them to treat patients with OSA. In addition, only one-quarter of the respondents used to refer their patients to a physician for a sleep-disordered diagnosis after noticing oral findings related to OSA. About one-third of the respondents attended continuing education courses on the management of OSA patients, and 79.33% were willing to attend a course on dental management of sleep-related oral diseases, as shown in Table 4.

**Table 4:** Frequency distribution and percentages of practice

Practice		N (%)
Have you ever asked your patient about his/her sleep history after observing the attrition of teeth in his mouth?	Always	75 (16.67%)
	Seldom	110 (24.44%)
	Never	265 (58.89%)
Have you ever screened a patient for OSA who has given a history of snoring?	Always	46 (10.22%)
	Seldom	118 (26.22%)
	Never	286 (63.56%)
Have you ever diagnosed an OSA patient?	Yes	86 (19.11%)
	No	364 (80.89%)
If yes, how many on average per year:	<5	45 (52.33%)
	>5	44 (47.67%)
Have you treated an OSA patient?	Yes	44 (9.78%)
	No	406 (90.22%)
If yes, how many on average per year:	<5	26 (59.09%)
	>5	17 (38.64%)
Have you ever fabricated any oral appliance for treating your patient with OSA?	Yes	34 (7.56%)
	No	416 (92.44%)
Have you referred your patient to a physician for a sleep-disordered diagnosis after noticing oral findings related to OSA?	Yes	113 (25.11%)
	No	337 (74.89%)
Have you ever attended any continuing education courses on the management of OSA patients?	Yes	149 (33.11%)
	No	301 (66.89%)
Would you be interested in attending a course on dental management of sleep-related oral diseases?	Yes	357 (79.33%)
	No	93 (20.67%)

## Discussion

Dentists frequently become the first contact point for many undiagnosed OSA patients, assisting in identifying, referring, and managing them [21]. Screening for OSA is made easier because of a patient's regular dental visits and the dentist's easy access to the upper airway. Therefore, the increasing frequency of undiagnosed patients and related hazards may be attributable to dentists' inadequate knowledge of OSA [22].

In this cross-sectional study, our findings showed that the knowledge score of Saudi dentists is below the optimal (score of 6), with a mean score of 3.09±1.36. The low level of knowledge of OSA among Saudi dentists was also reported by Alzahrani et al. [23] (Jeddah) and Swapna et al. (Riyadh) [24]. The gold-standard diagnosis and treatment modalities were unknown for about 51.6% and 63.56%, respectively. On the other hand, the majority of the participants in our study were aware of the common features and risk factors of OSA. Likewise, Alzahrani et al. found that most respondents knew common OSA signs and symptoms and could answer pertinent questions correctly [23]. However, other studies showed that the majority of dentists were not fully aware of the clinical and dental features of OSA. Swapna et al. reported that 53% of the participants were unaware of classic orofacial features noticed in OSA patients, including a deviation of nasal septal, macroglossia, tonsillar hypertrophy, long soft palate, large neck circumference, narrow palate, and maxillary and mandibular retrognathia [24]. Additionally, 83% were unaware of the Epworth sleepiness scale, and 86% did not know the Berlin questionnaire. They also showed that 20% of dentists did not have any idea if OSA patients suffer from severe snoring, and 63.5% believe that OSA can be seen in children [24]. Similar results were identified by Meenakshi et al., who showed that 81% of dental practitioners had no idea about the Epworth sleepiness scale [25]. Concerning the link between OSA and other disorders, research by Alansari et al. [26] found that dental interns in Saudi Arabia were under-informed on the correlation between OSA and diabetes (66%) and hypertension (58%). In order to increase OSA screening, facilitate its early diagnosis, prevent delayed referral, and lessen the resulting financial and health burden, dentists must be informed of the consequences of OSA.

Our findings demonstrated that 58.89% of the participants never asked their patients about sleep history after observing attrition of teeth in their mouth, and 63.56% never screened a patient for OSA who has given a history of snoring. Moreover, 80.89% and 90.22% of the participants never diagnosed or treated patients with OSA, respectively. Approximately 40% percent of participants in a previous Saudi investigation reported never having seen a patient with OSA. As few as 10% of dentists reported regularly seeing such patients [24]. These findings were quite comparable to those of the previous investigations, in which no more than 52% of the study group had ever seen these patients [25]. This information sheds light on the level of knowledge of this topic among dentists and their patients. Patients may feel uncomfortable discussing their snoring with a dentist because they assume the dentist cannot treat sleeping problems.

In our study, 32.67% of the participants did not know that dentists have a role in managing OSA patients. Similarly, Swapna et al. showed that 45% of Saudi dentists in Riyadh did not know that they have a role in screening and treating such cases [24]. Approximately 92.44% of our participants never used oral appliances to treat patients with OSA, while 25.11% preferred referring their patients to a physician for diagnosing and treating their condition. According to Swapna et al., the majority (58%) of participants referred OSA patients to a physician, whereas only 30% felt comfortable providing and treating using an oral appliance, and 12.5% decided to suggest lifestyle modifications. The application and outcomes of oral appliances in OSA have been widely studied in several research. But they failed to account for dentists, who play a crucial role in identifying OSA patients and providing appropriate recommendations or referrals [27]. Detecting and treating patients who may have OSA should be a standard part of any dentist's practice, regardless of their area of expertise. Dentists' preference for referring patients to specialist sleep specialists in order to reduce their legal exposure may indicate a lack of understanding of their jobs. Hence, increasing public understanding of their function and training them on OSA treatment might enhance the healthcare provided to patients with OSA. Orthodontists can play a crucial role in identifying and treating dental conditions that contribute to OSA [28]. By identifying those patients and referring them for a sleep study to confirm the diagnosis and then treating these conditions with oral appliances orthodontists can help improve patients' sleep quality and overall health. Orthodontists can work with other healthcare providers, such as sleep specialists and dentists, to provide comprehensive care to OSA patients and can be recognized as vital members of the healthcare team in treating OSA [29].

Our findings highlighted that the knowledge score was higher in specialists vs. GPs, orthodontics vs. restorative and esthetic dentistry specialists, post-graduate vs. undergraduate, those who previously diagnosed or treated cases with OSA vs. who did not, and in those who attended any course about OSA vs. who did not. On the other hand, there was no significant difference in the knowledge score based on age, sex, work sector, or years of experience. Similar findings were reported by Alzahrani et al., who found no significant differences between the mean attitude and knowledge scores based on practice sector, professional title, or sex [23]. They also mentioned that they could not find a significant difference between specialists and consultants in terms of OSA. These results are consistent with that of Jokubauskas et al., who concluded that general dentists and specialists were comparable in terms of their knowledge regarding OSA and ascribed this to the limited number of experts who responded and their advanced age [30]. Nevertheless, Vuorjoki-Ranta et al. showed that due to their higher levels of education, dental specialists were better informed about OSA [31].

While we recognize that our study has certain limitations, such as a low response rate and the absence of a detailed analysis of the categorical levels of knowledge due to the limited number of questions pertaining to OSA, it is important to note that these questions cover fundamental aspects of OSA-related knowledge.

## Conclusions

In conclusion, our study shows that dentists in Saudi Arabia lack fundamental understanding regarding OSA and their role in screening, diagnosing, and treating patients with OSA, highlighting the need to educate the dental fraternity. Dental GPs and specialists must be actively involved in preventing, diagnosing, and treating this life-threatening health issue.
